# Relationship between Marketing to Children on Food Labeling and Critical Nutrient Content in Processed and Ultra-Processed Products Sold in Supermarkets in Lima, Peru

**DOI:** 10.3390/nu12123666

**Published:** 2020-11-28

**Authors:** Daniella Torres-Schiaffino, Lorena Saavedra-Garcia

**Affiliations:** 1 School of Nutrition and Dietetics, Faculty of Health Sciences, Universidad Científica del Sur, Lima 15067, Peru; dtschiaffino@gmail.com; 2 CRONICAS Center of Excellence in Chronic Diseases, Universidad Peruana Cayetano Heredia, Lima 15074, Peru

**Keywords:** food labeling, marketing, M2K, ultra-processed food, children

## Abstract

Consumption of ultra-processed foods has increased alarmingly, representing a risk to children’s health. Different techniques in marketing to kids (M2K) used on food labels are influencing the purchasing decisions of these products. This study aims to provide useful information about M2K found in labeling of food products sold in a supermarket chain in Lima, Peru and to determine its relationship with critical nutrient content. This was an observational, correlational, descriptive study. Data were collected by photographing the front-of-pack (FoP) of 2747 product labels sold in three supermarkets in Lima, but only those that met all the inclusion criteria were evaluated (*n* = 1092). A relationship was found between the use of techniques in marketing to kids and the level of critical nutrient regarding saturated fat (PR = 0.56; CI95%: 0.52–0.63), total sugar (PR = 1.70; CI95%: 1.64–1.77), and sodium (PR = 1.05; CI95%: 1.03–1.07). Particularly with sugar, the presence of M2K is a risk factor. New regulatory policies for the use of these food labeling techniques should be implemented to improve children’s health at the population level.

## 1. Introduction

In recent years, the prevalence of overweight and obesity has increased alarmingly. Currently, the number of people with obesity worldwide represents twice the population with low weight [1], and among children, the prevalence of these indicators has also risen steadily. In 2016, according to the World Health Organization [2], 41 million children under five years old and 340 million children and adolescents between five and nineteen years old around the world were living with overweight or obesity, respectively. The situation in Peru is not completely different from the results of other countries. Based on the Peruvian National Household Survey (ENAHO, in Spanish), the prevalence of children under five years old living with overweight and obesity was 9.3%, 14.8% in children between five and nine years old, and 26% in adolescents in 2014 [3].

This situation is mainly due to an increase in the consumption of processed and ultra-processed foods with high levels of sugar, saturated fat, sodium, and calories [2,4], besides the decline in physical activity levels as a result of more sedentary lifestyle, transportation facilities, technology, and growing urbanization [2,4,5]. Moreover, it has been reported that high consumption of processed and ultra-processed foods as well as the greater exposure of children to advertisements are associated with increasing body fat [5].

Product packaging is the first physical contact between children and a product, but unlike adults, children are less likely to read the nutrition declaration, as they are influenced by other elements such as color, drawings, packaging shape, and characters when choosing a product [6]. Different studies demonstrate that the use of marketing can influence children’s attitudes and preferences regarding a product, as they are especially susceptible to non-textual messages [7,8,9,10,11,12]. For instance, a study carried out in Guatemala explored children’s preference and perception about the packaging of certain foods for the lunch box, using focus groups with children between 7 and 12 years old. Children chose their favorite product based on packaging, specifically on colors and drawings, which also influenced their perception of which product was the healthiest [9].

The Peruvian Law to Promote Healthy Eating for Children and Adolescents (Law No. 30021), whose priority is the protection of minors, was published in 2013 [13]. However, a detailed regulation with parameters for food advertising targeted at children has not yet been implemented.

Interestingly, according to the Pan American Health Organization, Peru has the largest growth in sales of ultra-processed products per capita in Latin America (15.6% from 179 to 207 kcal per capita/day) between 2009 and 2014 [14]. The new laws and regulations regarding the consumption of ultra-processed foods in children place Peru in a context in which it is crucial to take action to control the type of marketing techniques that industries use in the packaging of their products, especially those aimed to children.

The aim of this study is to provide information on the multiple techniques in marketing to kids found in labeling of products sold in supermarkets in Lima, Peru as well as to determine its relationship with the nutrition declaration of critical nutrient levels. Therefore, this study aims to generate information that contributes to decision-making processes in the formulation of policies that promote a healthier diet and generate information for the consumer.

## 2. Materials and Methods

### 2.1. Study Design and Settings

This was an observational, correlational, and descriptive study. Request letters were sent to access the two supermarket chains that have a nationwide presence in Peru and are aimed at different socioeconomic levels [15]. Of these two, only one agreed to give the permits. Once the permits were given, the stores that were closest to the investigation center were chosen (from each of the three socio-economic formats). The data were collected cross-sectionally between January and February 2018 in the three formats of the supermarket chain in Lima, Peru.

### 2.2. Variables

The variables of interest in this study were the techniques in marketing to kids (M2K) based on the categories proposed by Elliot C. [16], and the content and level of critical nutrients in processed and ultra-processed products categorized as “High” and “Acceptable” based on the parameters of Peruvian Law No. 30021, considering the following values in Table 1.

### 2.3. Sample Size and Data Sources

The visit to the three supermarkets was carried out during January and February 2018, and all barcoded products sold in the three supermarkets were photographed, taking as a reference the presentation of the product with the greatest exposure in the shelf (*n* = 2747). All sides of the packaging were photographed, and quality control for each photo was conducted at the end of its collection in order to verify that the text was legible as well as the images were recognizable.

Only those products with nutritional declaration were included (*n* = 1557). The declaration of nutrition information and the photographs were entered in the database program on food labeling “FLIP” (Food Label Information Program) developed by the University of Toronto [17]. The following data were recorded on the database program; product name, brand, weight, list of ingredients, and nutritional composition. Photos of each product were also uploaded.

For this study, products that did not declare any critical nutrient (saturated fat, sugar, or sodium) (*n* = 189), and those that required reconstitution for consumption (*n* = 150) were excluded. Subsequently, the remaining products were classified by food category in the same database program, based on the Table of Reference Amounts for Food in the Food and Drug Regulations of the Canadian Government [18]. Products belonging to categories that contain minimally processed foods, ingredients, or nutritional supplements were also excluded (*n* = 101) from the analysis. These categories were oils, fresh fish and seafood, legumes, mixed dishes, potatoes and tubers, salads, condiments, and vegetables.

Finally, the nutritional information declared in the labeling was validated, using the Atwater System calculation with an error range of <20%. This validation consists of determining the caloric content of a product from its macronutrients (carbohydrates, proteins, and total fat), then multiplying it by the Atwater constants and comparing it to the total energy declared on the package. Those products with inconsistent values of its nutrients (*n* = 25) were excluded. A remaining total of 1092 products were assessed in this study.

### 2.4. Procedures

The determination of critical nutrient content was carried out by observing the nutrition facts table of the product, and it was classified in “High” or “Acceptable” content of saturated fat, total sugar, and sodium, using the parameters of the Manual of advertising warnings of the regulation of Peruvian law No. 30021. [13].

The determination of M2K on the labeling was conducted by observing the FoP of each product, and the presence of the following techniques was sought; use of characters, drawings, celebrities, athletes, children images, gifts, collectible packaging, playful packaging, contests, animated letters, or special colors and flavors [17,19]. If the same type of technique was repeated two or more times in the same product, it was considered as one.

### 2.5. Bias

In the following study, it was sought to assess all the processed and ultra-processed products available in the market. For that reason, photographs were collected in three nationwide supermarkets in Lima, known for serving three specific but different socioeconomic sectors (high, medium, and low sectors).

Only one format of each product was photographed, but it was the one with the greatest exposure at the point of sale.

To avoid repeatedly capturing food products, photographs of all the products found in the first supermarket were taken. Then, only after finishing with the first supermarket we moved on to the next one, and only the products that had not been photographed before were captured. On the other hand, the Flip software was able to detect repeated barcodes when entering the ID, reducing the probability of repeated testing.

The database was cleaned up, and the information of the nutritional composition declared on the labeling was validated, using the Atwater System calculation with an acceptable margin of error of <20%. In order to exclude products that may have incorrectly declared nutrition information, it was decided to exclude those products with incoherent nutritional information and those with incoherent values of critical nutrients in case they exceeded the number of total macronutrients.

To avoid possible observer bias, a double coding of the marketing strategies was carried out by two previously trained nutritionists. The discrepancies found between the two experts were solved by verifying the images together.

### 2.6. Data Analysis

First, the normality of continuous quantitative variables was evaluated through the Shapiro-Wilk test. As no normal distribution was found, these variables were expressed through descriptive statistics using medians and interquartile ranges (IQR).

The categorical variables were presented through frequencies and percentages.

Pearson’s chi-square test was used to determine the relationship between variables, marketing techniques targeted at children and the content of critical nutrients among all processed and ultra-processed foods. The analysis for each food or food category separately was not feasible, since there were some categories with very few elements to be analyzed. To determine the strength of this relationship, the prevalence ratio (PR) was also determined.

### 2.7. Ethics

Ethical approval for this study was obtained from the Institutional Review Board of Universidad Peruana Cayetano Heredia (project 201241) and the Ethics Committee of the Universidad Científica del Sur, with the supermarket chain permission to collect information. This study is based on the information declared on labels of packages, and therefore does not represent a risk for humans.

## 3. Results

A total of 1092 (70%) products were included in the analysis of this study, 33% of which (*n* = 358) were from “sugars and sweets” category, 13% (*n* = 146) were from the “beverages” category, and 12% (*n* = 133) from the “cereals and other grain products” category. All products specified at least one of the three critical nutrients evaluated on the label (saturated fat, total sugar, and sodium).

### 3.1. Marketing Techniques

Only five of the eight evaluated techniques were evidenced on the FoP (allusions to fun or games, attractive letters or graphics for children, use of characters, toys or prizes and use of colors, flavors, or unusual shapes). The strategies such as contests or raffles for children, discounts or coupons, and games for children were not evidenced; therefore, they were withdrawn from the evaluation. It was determined that 12% (*n* = 136) of products contained at least one M2K technique on the packaging. In addition, the most used technique was the use of letters or graphics that were appealing to children, representing 88% (*n* = 119) of the total (e.g., animated or cartoonish letters), followed by the use of unusual colors, flavors, or shapes, representing 68% (*n* = 93) of the total (e.g., containers with collectible shapes and fantasy flavors) and the use of characters, representing 67%(*n* = 91) of the total (e.g., cartoons of animals and fictional characters).

On the other hand, M2K was evidenced in 7 of 15 categories. The following categories did not demonstrate the use of these strategies in its products; “bakery products”, “desserts”, “dessert fillings”, “fruit juices”, “miscellaneous”, “nuts and seeds”, “butter and margarine”, and “sauces and dressings”. Table 2 indicates categories with the greatest use of M2K. Among them, 100% (*n* = 6) of products belonging to the “foods for children under 4 years old” category use M2K techniques, 83% (*n* = 5) of which use more than one technique at the same time. Moreover, 23% of the “cereals and other grain products” category use M2K technique (*n* = 30), 100% of which (*n* = 30) apply more than one technique. Finally, 22% (*n* = 77) of “sugars and sweets” category use M2K techniques, 95% (*n* = 73) of which use more than one marketing technique in FoP.

### 3.2. Content and Classification of Critical Nutrients

Table 3 shows the content of critical nutrients by category in processed and ultra-processed foods sold in Peru. Categories with the highest content of saturated fats were “butter” (30 g/100 g; IQR = 17.8–50.0), “sugar and sweets” (10.8 g/100 g; IQR = 5.5–17.2), and “dessert fillings” (7.5 g/100 g; IQR = 4.4–12.0). Total sugar was higher in “dessert fillings” category (47g/100g; IQR = 34.2–52.0), “sugar and sweets” category (43 g/100 g; IQR = 30.0–55.0), and “miscellaneous” category (35.2 g/100 g; IQR = 9.3–60.0). However, it should be noted that the categories of “cereals and other grain products” (14.2 g/100 g; IQR = 3.4–29.1) and “foods for children under 4 years old” (8.3 g/100 g; IQR = 6.0–10.3) are also among the products with the highest total sugar content. The categories with the highest sodium content per 100g of product were “meats and sausages” (929.6 mg/100 g; IQR = 309.5–1617.5), “sauces and dressings” (774 mg/100 g; IQR = 365.6–1178.3), and “butter and margarine” (760.7 mg/100 g; IQR = 450.0–892.8).

As shown in Table 4, 65% (*n* = 709) of the evaluated products (*n* = 1092) had a high value of at least one critical nutrient. Thirty-three percent (*n* = 360) of the total were classified as high in saturated fat, 48% (*n* = 519) of the total were classified as high in total sugar, and 8% (*n* = 89) of the total was considered high in sodium.

One-hundred percent (*n* = 18) of “butter and margarine” category, 94% (*n* = 15) of “dessert fillings” category, and 91% (*n* = 324) of “sugar and sweets” category had the highest proportion of products high in at least one critical nutrient. Regarding saturated fats, 94% (*n* = 17) of “butter and margarine” category, 66% (*n* = 235) of “sugar and sweets” category and 58% (*n* = 11) of “nuts and seeds” category were the ones that obtained the highest values. Furthermore, 82% (*n* = 294) of “sugar and sweets” category as well as 81% (*n* = 13) of “fillings for desserts” category and 67% (*n* = 4) of “foods for children under 4 years old” category obtained the highest values for total sugar level. Regarding sodium, 51% (*n* = 41) of “sauces and dressings” category, 50% (*n* = 9) “butter and margarine” category, and 50% (*n* = 4) of “meats and sausages” category were the ones with the highest proportion of products with high levels of this nutrient.

Thirty-five percent (*n* = 383) of all products (*n* = 1092) contain the three critical nutrients at adequate levels, 41% (*n* = 451) contain only one critical nutrient with high levels, and 24% (*n* = 257) contain two high critical nutrients, but there was only one product (0%) with high levels of the three of them: saturated fat, total sugar and sodium.

### 3.3. Relationship between Marketing to Children on Food Labeling and Critical Nutrient Content in Processed and Ultra-Processed Products

A significant relationship was discovered between the use of M2K on food labeling and the critical nutrient content in both total sugar (PR = 1.70; CI95%: 1.64–1.77) and sodium (PR = 1.05; CI95%: 1.03–1.07). The use of marketing techniques was a risk factor about the content of these nutrients. However, the strength of the association regarding the sodium was low. On the other hand, regarding saturated fat (PR = 0.56; CI95%: 0.52–0.63), a relationship was found between the variables, but the use of marketing techniques would act rather as a protective factor regarding the content of this nutrient in processed and ultra-processed products sold in Lima, Peru.

## 4. Discussion

### 4.1. Main Results

From the evaluation of a total of 1092 processed and ultra-processed products collected in three supermarkets in Lima, Peru, it is clear that there is a relationship between the use of M2K techniques on product labeling and the content of critical nutrients. Among the most relevant results, we obtain that categories that use most of the marketing techniques are precisely those of “foods for children under 4 years old”, as well as “cereals and other grain products”, which are also among the categories with the highest total sugar content. In addition, more than half of the products are considered “High” in at least one critical nutrient.

### 4.2. Comparison with Previous Studies

The main results of this study are consistent with other studies previously performed on the market [20,21,22,23,24,25,26]. Elliot C [21] found that there is indeed a significant relationship between the use of promotional techniques on packaging and the nutritional content of products, proving that 88.8% of products that used these strategies were classified as low nutritional quality products. [20] In another research, a similar result was found: 86.9% of products targeted at children with high critical nutrient content [22]. Likewise, in both cases, the use of appealing letters and fonts was detected as the main strategy [21,22], which is consistent with the result of this investigation.

This study also demonstrated that more than a half of the products are high in at least one critical nutrient, being sugar the most common nutrient with the highest level. According to Gamboa T and collaborators [23], a significant high level of sugar and sodium was found in those products that used marketing techniques in Costa Rica. Additionally, it is mentioned that those categories with the highest declared total sugar levels are confectionery products and breakfast cereals [27].

An unexpected result was considering saturated fat as a protective factor against the use of M2K techniques. It is important to mention that less than 10% of the products considered high in saturated fat use M2K, and these products were entirely corresponding to “Salty snacks” and “Sugar and sweets” categories. As these products are a minority, it influences the determination of saturated fat as a protective factor, as the highest proportion of products high in saturated fat, in addition to the aforementioned categories, corresponded to categories such as “Butter and Margarine”, “Nuts and seeds”, “Sauces and dressings”, and “Meat and sausages “, which, by nature, do not focus on children as the main target.

Regardless of the use of promotional techniques, various studies agree that the majority of processed and ultra-processed products found in the markets exceed the established parameters for the critical nutrient content [22,24,25,28]. Because of this, these results are important to help in decision-making processes for new guidelines and control methods for the use of promotional strategies aimed to children.

### 4.3. Results Interpretation

The results of this study are expected. Companies use these marketing techniques intending to capture the attention of children and attract them to a certain product. Rachel S et al. [6] as well as other researchers [8,9,10,11,12,29,30,31] emphasize that the use of marketing could influence children’s attitudes and preferences regarding a product, since children are especially susceptible to non-text messages. Unlike other studies, although a relationship was found between critical nutrients and marketing techniques, when analyzing the strength of association of each nutrient, sodium is the weakest (PR = 1.05; CI95%: 1.03–1.07). This may be due to, unlike other studies, Peru uses under 800 mg/100 g values as an acceptable parameter for sodium according to Law No. 30021 [13]. Consequently, products evaluated as high in sodium in other countries would be considered acceptable in Peru. Gamboa T [23], in his research in Costa Rica, used 300 mg/100 g as a parameter for sodium and obtained a significant relationship between sodium and the marketing strategies that were used [24].

Regarding the differences found in terms of the percentage of products considered high in critical nutrients according to the labeling, it is important to take into consideration that in Peru the use of a nutrition facts table in food labeling is not mandatory, and not all products declare all of the three critical nutrients evaluated.

Finally, in other studies, additionally to M2K techniques, different health-related claims were evaluated, and claims such as “source of”, “low in”, and “free of” were observed on the FoP of products with less nutritional quality [21,22]. This is an item that was not evaluated in this study; however, it would be interesting to take it into account for further evaluation.

### 4.4. Public Health Implications

The results show that the offered products, especially the categories targeted at children, have a high presence of marketing to this population and, at the same time, a high level of critical nutrients. This is a call to the authorities since there is a Peruvian law to promote Healthy Eating for Children and Adolescents (Law No. 30021) published in 2013 [13], which has the protection of minors as a priority. However, until today, the article No. 8 about advertising control to promote healthy eating has not been clearly regulated. Although there is this article that refers to advertising and communication, there is no specific and detailed manual with guidelines and parameters to follow for the proper execution and control over the promotional techniques used in products aimed to children. For that reason, as presented in this study, children are still exposed to elements that promote an unhealthy food environment.

On the other hand, it is important to implement actions to promote children’s and adolescent´s education on healthy food consumption in order to support other food environment interventions. According to the Guidelines of the Peruvian Ministry of Health for the Promotion and Protection of Healthy Eating in Educational Institutions, it is proposed to implement policies, plans, or pedagogical resources that promote nutritional education, healthy eating, and physical activity with an intercultural approach, in conjunction with the Ministry of Education [32]. Some initiatives, such as the limitation of the available products in scholar kiosks, the supervision of the preparations by a professional nutritionist in cafeterias, and the control of the products displayed in vending machines, exist, but these initiatives have not yet been implemented [33].

### 4.5. Strengths and Limitations

Regarding the products chosen for evaluation, the product format with the highest exposure on the shelf was used. Not all formats of the same product were included to be observed in this study. Furthermore, the nutritional information was taken from the label of the sold products, of which 1190 out of 2747 did not declare any information. Products for sale in small markets and other selling points were evaluated; however, among the main strengths of this study, we considered that the majority of processed and ultra-processed products in Peru are available in supermarkets. Likewise, the three supermarkets chosen for data collection encompasses three different economic sectors (high, medium, and low). Although the sample was taken in Lima, it is a supermarket chain with presence throughout the country. Finally, this study creates a basis for new lines of research on the subjects as well as a call to action towards public entities.

## 5. Conclusions

A significant relationship was found between the use of M2K techniques and critical nutrient levels of saturated fat, total sugar, and sodium. This information will be useful to consumers’ purchase decisions in the future. As a country, Peru has the largest growth in sales of ultra-processed products per capita in Latin America between 2009 and 2014, according to the Pan American Health Organization, new regulatory policies for the use of these techniques on food labeling should be implemented to complement, and strengthen the Law of Healthy Eating in order to improve children’s health at population level.

It is recommended to adopt measures and apply these marketing strategies on the promotion of natural and high nutritional value foods to make them more appealing to children.

## Figures and Tables

**Table 1:** Saturated fat, total sugar, and sodium parameters according to Peruvian Law No. 30021. ^(a)^.

Technical Parameters	Considered as “HIGH”
Saturated Fat (solids)	Greater than or equal to 6 g/100 g
Saturated Fat (liquids)	Greater than or equal to 3 g/100 mL
Total sugar (solids)	Greater than or equal to 22.5 g/100 g
Total sugar (liquids)	Greater than or equal to 6 g/100 mL
Sodium (solids)	Greater than or equal to 800 mg/100 g
Sodium (liquids)	Greater than or equal to 100 mg/100 mL

^(a)^ Parameters according to Manual of advertising warnings of the regulation of the Peruvian law No. 30021, Law to Promote Healthy Eating for Children and Adolescents—First phase [13].

**Table 2:** Presence of marketing to kids in the labeling of products by food category.

Marketing to Kids (M2K)			
Food Categories ^(a)^	n (%) Products with M2K ^(b)^	n (%) Products with M2K with a Single Technique in FOP ^(c)^	n (%) M2K Products with More than One Technique in FOP
Beverages	7 (5)	6 (86)	1 (14)
Cereals and other grain products	30 (23)	0 (0)	30 (100)
Dairy products and substitutes	7 (10)	0 (0)	7 (100)
Salty snacks	3 (3)	0 (0)	3 (100)
Sugar and sweets	77 (22)	4 (5)	73 (95)
Food for children under 4 years old	6 (100)	1 (17)	5 (83)
Meat and sausages	1 (13)	0 (0)	1 (100)

^(a)^ Categories according to Nutrition Labelling—Table of Reference Amounts for Food, Government of Canada, Health Canada [18]. ^(b)^ M2K: Marketing to kids. ^(c)^ FoP: Front of pack.

**Table 3:** Critical nutrient content ^(a)^ per 100 g/mL in processed and ultra-processed foods by food category ^(b)^.

	Saturated Fat (g)		Total Sugar (g)		Sodium (mg)	
Food Category (*n* = 15)	n (%)	Median (IQR)	n (%)	Median (IQR)	n (%)	Median (IQR)
Bakery products	48 (96)	1.1 (0.0–3.3)	48 (96)	4.7 (3.1–7.8)	38 (76)	330.6 (67.4–454.5)
Beverages	53 (36)	0.0 (0.0–0.0)	143 (97)	7.1 (4.6–10.5)	130 (89)	10.1 (5.3–36.0)
Cereals and other grain products	111 (83)	0.3 (0.0–1.0)	132 (99)	14.2 (3.4–29.1)	102 (76)	124.7 (0.0–363.6)
Dairy products and substitutes	32 (45)	1.0 (0.1–1.5)	32 (45)	9.4 (5.8–13.9)	30 (42)	48.0 (43.0–57.7)
Desserts	35 (87)	2.3 (1.3–5.9)	18 (45)	14.0 (11.7–19.2)	37 (92)	39.0 (14.7–55.0)
Dessert fillings	9 (56)	7.5 (4.4–12.0)	13 (81)	47.0 (34.2–52.0)	14 (87)	107.8 (55.5–133.3)
Natural fruit juice	8 (57)	0.0 (0.0–0.0)	13 (92)	4.0 (2.8-11.0)	13 (92)	9.5 (5.0–16.8)
Miscellaneous	26 (60)	0.0 (0.0–1.8)	42 (97)	35.2 (9.3–60.0)	37 (86)	0.0 (0.0–58.8)
Nuts and seeds	19 (100)	6.6 (5.0–7.1)	16 (84)	4.5 (3.4–7.3)	16 (84)	310.1 (53.3–422.0)
Salty snacks	89 (97)	6.6 (2.8–9.1)	86 (94)	3.3 (0.0–6.6)	75 (82)	514.2 (380.0–772.7)
Sugar and sweets	314 (87)	10.8 (5.5–17.2)	336 (93)	43.0 (30.0–55.0)	333 (93)	104.2 (37.5–236.1)
Food for children under 4 years old	6 (100)	1.7 (1.3–1.8)	6 (100)	8.3 (6.0–10.3)	6 (100)	50.0 (48.0–52.0)
Butter and Margarine	17 (94)	30.0 (17.8–50.0)	18 (100)	0.0 (0.0–0.0)	5 (27)	760.7 (450.0–892.8)
Meat and sausages	5 (62)	12.5 (3.2–12.7)	8 (100)	0.8 (0.0–2.3)	3 (37)	929.6 (309.5–1617.5)
Sauces and dressings	57 (71)	1.7 (0.0–6.2)	71 (88)	5.0 (1.0–10.0)	55 (68)	774.0 (365.6–1178.3)

^(a)^ Data expressed through medians and interquartile ranges (IQR) (25-75 percentiles). ^(b)^ Categories according to Nutrition Labelling—Table of Reference Amounts for Food, Government of Canada, Health Canada [18].

**Table 4:** Critical nutrient level ^(a)^ in processed and ultra-processed products by food category ^(b)^.

	Saturated Fat		Total Sugar		Sodium		Total per Category	
Food Category (*n* = 15)	High n (%)	Acceptable n (%)	High n (%)	Acceptable n (%)	High n (%)	Acceptable n (%)	High n (%)	Acceptable n (%)
Bakery products	6 (12)	44 (88)	6 (12)	44 (88)	1 (2)	49 (98)	11 (22)	39 (78)
Beverages	0 (0)	146 (100)	86 (59)	60 (41)	0 (0)	146 (100)	86 (59)	60 (41)
Cereals and other grain products	1 (1)	132 (99)	36 (27)	97 (73)	7 (5)	126 (95)	40 (30)	93 (70)
Dairy products and substitutes	0 (0)	70 (100)	15 (21)	55 (79)	0 (0)	70 (100)	15 (21)	55 (79)
Desserts	10 (25)	30 (75)	24 (60)	16 (40)	0 (0)	40 (100)	26 (65)	14 (35)
Desserts fillings	5 (31)	11 (69)	13 (81)	3 (19)	1 (6)	15 (94)	15 (94)	1 (6)
Natural fruit juice	0 (0)	14 (100)	6 (43)	8 (57)	1 (7)	13 (93)	7 (50)	7 (50)
Miscellaneous	5 (12)	38 (88)	22 (51)	21 (49)	1 (2)	42 (98)	28 (65)	15 (35)
Nuts and seeds	11 (58)	8 (42)	1 (5)	18 (95)	1 (5)	18 (95)	13 (68)	6 (32)
Salty snacks	50 (55)	41 (45)	4 (4)	87 (96)	21 (23)	70 (77)	63 (69)	28 (31)
Sugar and sweets	235 (66)	123 (34)	294 (82)	64 (18)	2 (1)	356 (99)	324 (91)	34 (9)
Food for children under 4 years old	0 (0)	6 (100)	4 (67)	2 (33)	0 (0)	6 (100)	4 (67)	2 (33)
Butter and Margarine	17 (94)	1 (6)	0 (0)	18 (100)	9 (50)	9 (50)	18 (100)	0 (0)
Meat and sausages	3 (38)	5 (63)	0 (0)	8 (100)	4 (50)	4 (50)	4 (50)	4 (50)
Sauces and dressings	17 (21)	63 (79)	8 (10)	72 (90)	41 (51)	39 (49)	55 (69)	25 (31)
Total per critical nutrient	360 (33)	732 (67)	519 (48)	573 (52)	89 (8)	1003 (92)	709 (65)	383 (35)

^(a)^ Classification according to parameters of Peruvian Law No. 30021 to promote healthy eating for children and adolescents—First phase [13]. ^(b)^ Categories according to Nutrition Labelling—Table of Reference Amounts for Food, Government of Canada, Health Canada [18].
